# Study on structural geometry and dynamic property of [NH_3_(CH_2_)_5_NH_3_]CdCl_4_ crystal at phases I, II, and III

**DOI:** 10.1038/s41598-022-08246-5

**Published:** 2022-03-11

**Authors:** Ae Ran Lim, Yong Lak Joo

**Affiliations:** 1 Department of Carbon Convergence Engineering, Jeonju University, Jeonju, 55069 Korea; 2 Department of Science Education, Jeonju University, Jeonju, 55069 Korea; 3 Robert Fredrick Smith School of Chemical and Biomolecular Engineering, Cornell University, Ithaca, NY 14853 USA

**Keywords:** Condensed-matter physics, Materials chemistry

## Abstract

Organic–inorganic hybrid perovskites can potentially be used in electrochemical devices, such as batteries and fuel cells. In this study, the structure and phase transition temperatures of the organic–inorganic material [NH_3_(CH_2_)_5_NH_3_]CdCl_4_ crystal were confirmed by X-ray diffraction and differential scanning calorimetry. From the nuclear magnetic resonance results, the crystallographic configurations of ^1^H, ^13^C, and ^14^N in the cation changed at temperatures close to T_C1_ (336 K), whereas that of ^113^Cd in the anion shows significant changes at temperatures close to T_C1_ and T_C2_ (417 K). The activation energy, E_a_, values for ^1^H and ^13^C obtained from the spin–lattice relaxation time, T_1ρ_, below and above T_C1_ were evaluated, where the E_a_ value for ^13^C was more flexible at low temperatures than at high temperatures. In addition, the effect on molecular motion was effective at high temperatures. The phase transition at 336 K was associated with the change in the N–H···Cl bond due to the change in the coordination geometry of Cl around Cd in the CdCl_6_ anion. On the other hand, the phase transition at 417 K was related to the ferroelastic phase transition attributed to the twin domains.

## Introduction

Recently, many studies with the development of functional materials are being conducted on organic–inorganic hybrid perovskite materials. The organic–inorganic hybrid crystal [NH_3_(CH_2_)_*n*_NH_3_]*BX*_4_ (*n* = 2, 3, 4, …), where *B* is a transition metal, such as Mn, Cd, Fe, Cu …, and *X* is a halogen ion, crystallizes perovskite-type layer structures^1–12^. The organic part of the hybrid complex determines the optical properties and structural flexibility, whereas the inorganic part affects the mechanical and thermal properties^13^. The properties and structural phase transitions of organic–inorganic hybrid compounds are affected by their structures and the interactions between cation and anion^12^. For chains in which *n* $\gg$ 4, structural rearrangement by conformational changes in the chains becomes important. An interesting group of hybrid compound is the perovskite-type layer [NH_3_(CH_2_)_5_NH_3_]CdCl_4_ (pentylenediammonium cadmium tetrachloride) containing a [NH_3_(CH_2_)_5_NH_3_] cation and a two-dimensional (2D) layered CdCl_6_ anion. [NH_3_(CH_2_)_5_NH_3_]CdCl_4_ has two structural phase transitions at temperatures near 337 K (T_C1_) and 417 K (T_C2_)^14,15^. It exhibits an unusual phase sequence, in which the phase that is stable at high temperatures exhibits the lowest symmetry.

The phase sequence in the following way^16^

Phases III (below 337 K) and II (above 337 K) are orthorhombic with the space groups *Pnam* and *Imam*, respectively. The lattice constants in phase III (at 293 K) are *a* = 7.330 Å, *b* = 7.504 Å, *c* = 23.862 Å, and Z = 4, while the unit cell parameters in phase II (at 353 K) are *a* = 7.376 Å, *b* = 7.561 Å, *c* = 23.555 Å, and Z = 4. The high-temperature phase I is monoclinic, and the unit cell parameters at 433 K are *a* = 7.516 Å, *b* = 7.563 Å, *c* = 11.22 Å, and β = 98.15° with the space group *C12/m*_*1*_. The [NH_3_(CH_2_)_5_NH_3_] organic chains are arranged along the longest *c*-axis. The Cd octahedra is located the edge to form a 2D network, and the diammonium cations are connected to CdCl_6_ octahedra by hydrogen bonds. In the inorganic layers, the structural geometries around the Cd atoms are described as distorted octahedra. These hybrid perovskite materials have potential applications in various electrochemical devices, such as batteries and fuel cells^17–25^.

The synthesis and characterization of [NH_3_(CH_2_)_5_NH_3_]CdCl_4_ were first discussed by Kind et al.^26^, where the structural phase transitions were studied using ^35^Cl and ^2^D nuclear magnetic resonance (NMR), birefringence, dilatation measurements, and optical domain investigations. Negrier et al.^15^ evaluated the crystal structures via X-ray diffraction (XRD) and Raman scattering experiments at 293 K and 353 K. Our group has also recently reported the effects of ^13^C length in the cation of [NH_3_(CH_2_)_2_NH_3_]CdCl_4_, [NH_3_(CH_2_)_3_NH_3_]CdCl_4_, and [NH_3_(CH_2_)_4_NH_3_]CdCl_4_ crystals on the thermal and structural dynamic properties^13^. Meanwhile, a lot of research has been done on the electrical and conductive properties of this type of compound^16,27–30^.

Here, the crystal structures, thermodynamic properties, and ferroelastic domain walls of [NH_3_(CH_2_)_5_NH_3_]CdCl_4_ were investigated. The roles of cations and anions in the [NH_3_(CH_2_)_5_NH_3_]CdCl_4_ single crystal were discussed, and the chemical shifts and spin-lattice relaxation time, T_1ρ_, with increasing temperature were measured using ^1^H magic angle spinning (MAS) NMR, ^13^C MAS NMR, and static ^14^N NMR to identify the roles of the [NH_3_(CH_2_)_5_NH_3_] cation. Furthermore, the ^113^Cd MAS NMR chemical shifts were recorded to evaluate the coordination geometry of the CdCl_6_ anion. The results would provide insights into the physicochemical properties of [NH_3_(CH_2_)_5_NH_3_]CdCl_4_ crystals, facilitating their various applications in the future.

## Methods

A saturated aqueous solution containing NH_2_(CH_2_)_5_NH_2_·2HCl and CdCl_2_ was gradually evaporated at 300 K to grow single crystals of [NH_3_(CH_2_)_5_NH_3_]CdCl_4_. Colorless single crystals measuring approximately 7 mm × 3 mm × 2 mm were grown for approximately 2–3 weeks in the thermostat.

The structures of the [NH_3_(CH_2_)_5_NH_3_]CdCl_4_ crystals at 298 K were analyzed using an XRD system. The lattice parameter and space group was considered by single-crystal XRD at the Seoul Western Center of the Korea Basic Science Institute. Experiments were performed in the same manner as before^31^.

Differential scanning calorimetry (DSC) (TA, DSC 25) experiments were carried out at a heating rate of 10 K/min from 190 to 550 K in N_2_ gas. Thermogravimetric analysis (TGA) and differential thermal analysis (DTA) curves were obtained using a thermogravimetric analyzer (TA Instrument) with the same heating rate as in DSC from 300 to 973 K in N_2_ gas. In addition, the domain patterns were observed using an optical polarizing microscope within the temperature range of 300 to 450 K, where the prepared single crystals were placed on the plate with the temperature sensor of a Linkam THM-600.

NMR spectra of the [NH_3_(CH_2_)_5_NH_3_]CdCl_4_ crystals were performed using a Bruker 400 MHz Avance II+ solid-state NMR spectrometer in the same facility. The Larmor frequencies for ^1^H and ^13^C MAS NMR experiments were 400.13 and 100.61 MHz, respectively. In MAS NMR experiment, the spinning speed was set to 10 kHz to minimize sideband. And tetramethylsilane (TMS) was used as a standard material to obtain accurate NMR chemical shift. The experimental method to obtain the T_1ρ_ values for ^1^H and ^13^C was used in the same way as the previously reported method^13^. And, static ^14^N NMR and ^113^Cd MAS NMR spectra were recorded at Larmor frequencies of 28.90 and 88.75 MHz, respectively. ^14^N and ^113^Cd chemical shift measurements were performed using NH_4_NO_3_ and CdCl_2_O_8_·6H_2_O as standard materials.

## Experimental results

### Crystal structure

**Figure 1:**
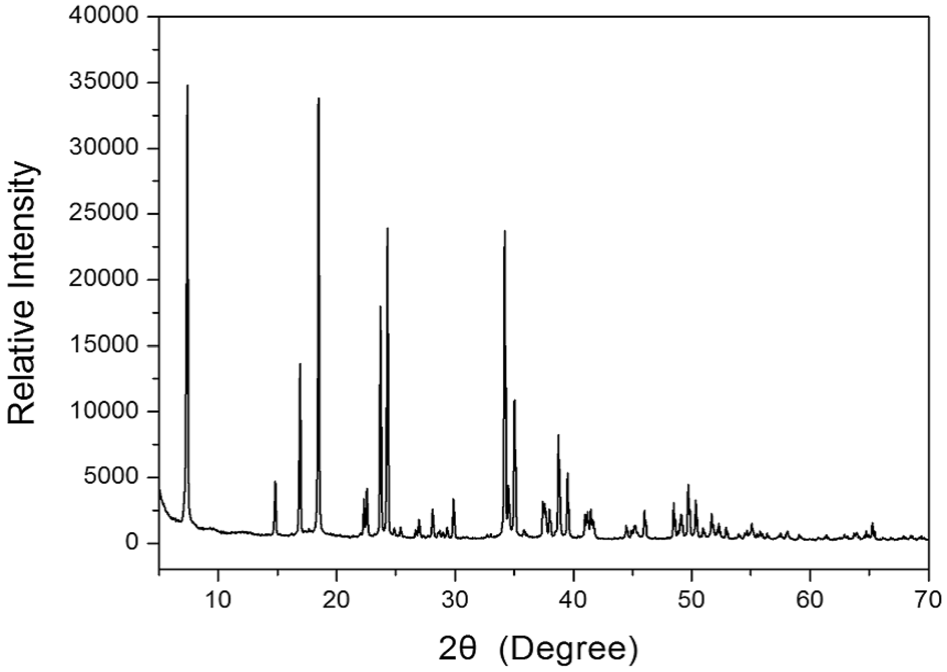
XRD powder pattern of the [NH_3_(CH_2_)_5_NH_3_]CdCl_4_ crystal at 298 K.

The powder XRD pattern of the [NH_3_(CH_2_)_5_NH_3_]CdCl_4_ crystal at 298 K is shown in Fig. 1. And, the lattice constants analysized from the X-ray crystal diffraction were determined to be *a* = 7.3292 ± 0.002 Å, *b* = 7.5058 ± 0.002 Å, and *c* = 23.9376 ± 0.006 Å with the space group *Pnam*; this is consistent with the previously reported results^14,15^.

### Phase transition temperature, thermal property, and ferroelastic twin domain

**Figure 2:**
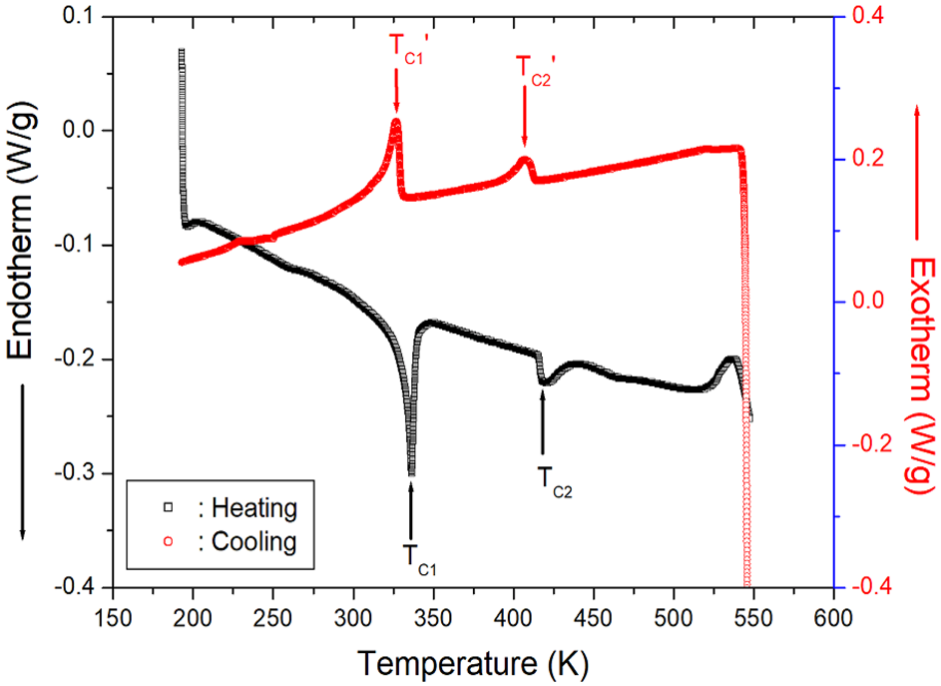
DSC curves of [NH_3_(CH_2_)_5_NH_3_]CdCl_4_ during heating and cooling.

The DSC curves of the [NH_3_(CH_2_)_5_NH_3_]CdCl_4_ crystal at a heating and cooling rate of 10 K/min in N_2_ gas are presented in Fig. 2. Two endothermic peaks were observed at 336 K (T_C1_) and 418 K (T_C2_) during heating, whereas two exothermic peaks were recorded at 327 K (T_C1_′) and 407 K (T_C2_′) during cooling. The phase transition enthalpy on heating is 3.17 kJ/mol at 337 K and 0.55 kJ/mol at 417 K, respectively. On the other hand, previous studies reported endothermic peaks at 337 K and 417 K during heating and at 336 K and 407 K during cooling^14,15^.

**Figure 3:**
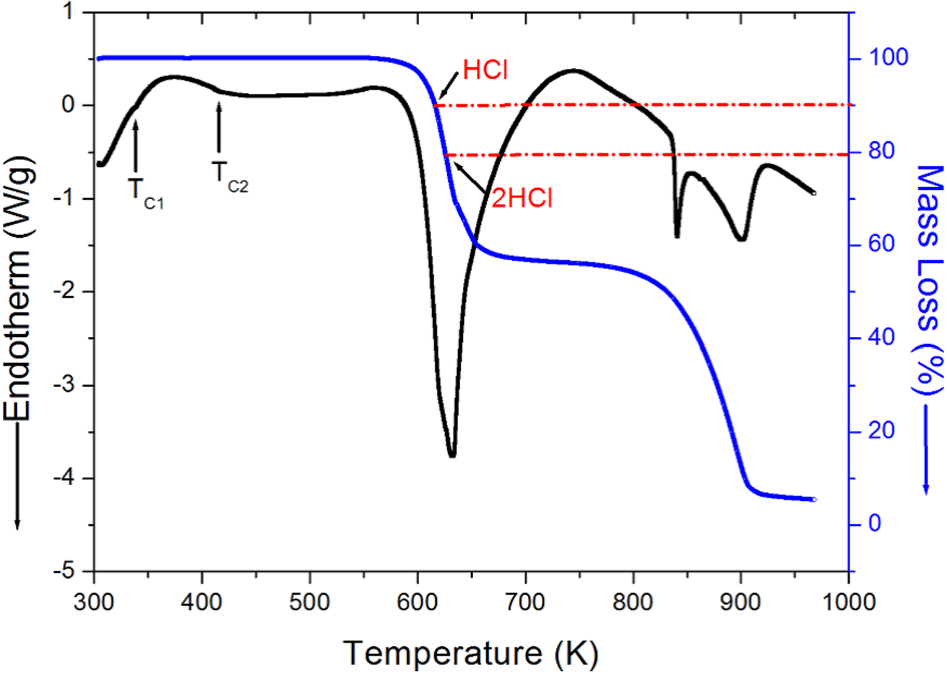
TGA and DTA curves of [NH_3_(CH_2_)_5_NH_3_]CdCl_4_.

To determine the preliminary thermal characteristics, including the structural phase transitions, TGA and DTA results were conducted at the same heating rate as the DSC experiment. Based on the TGA and DTA curves shown in Fig. 3, the crystal exhibited excellent stability up to approximately 600 K. The small inflection points observed at temperatures near 336 K and 417 K in the DTA curve were coincides with the two phase transition temperatures obtained from the DSC results, suggesting that the molecular weight of [NH_3_(CH_2_)_5_NH_3_]CdCl_4_ decreased at increasing temperatures. The amount of crystal remaining in the solid state was evaluated from the molecular weights. The 10% and 20% weight losses of the crystal at temperatures of about 617 K and 626 K were attributed to the decomposition of HCl and 2HCl, respectively. On the other hand, the weight loss at approximately 800 K and 900 K shown in Fig. 3 was observed 46% and 87%, respectively.

**Figure 4:**
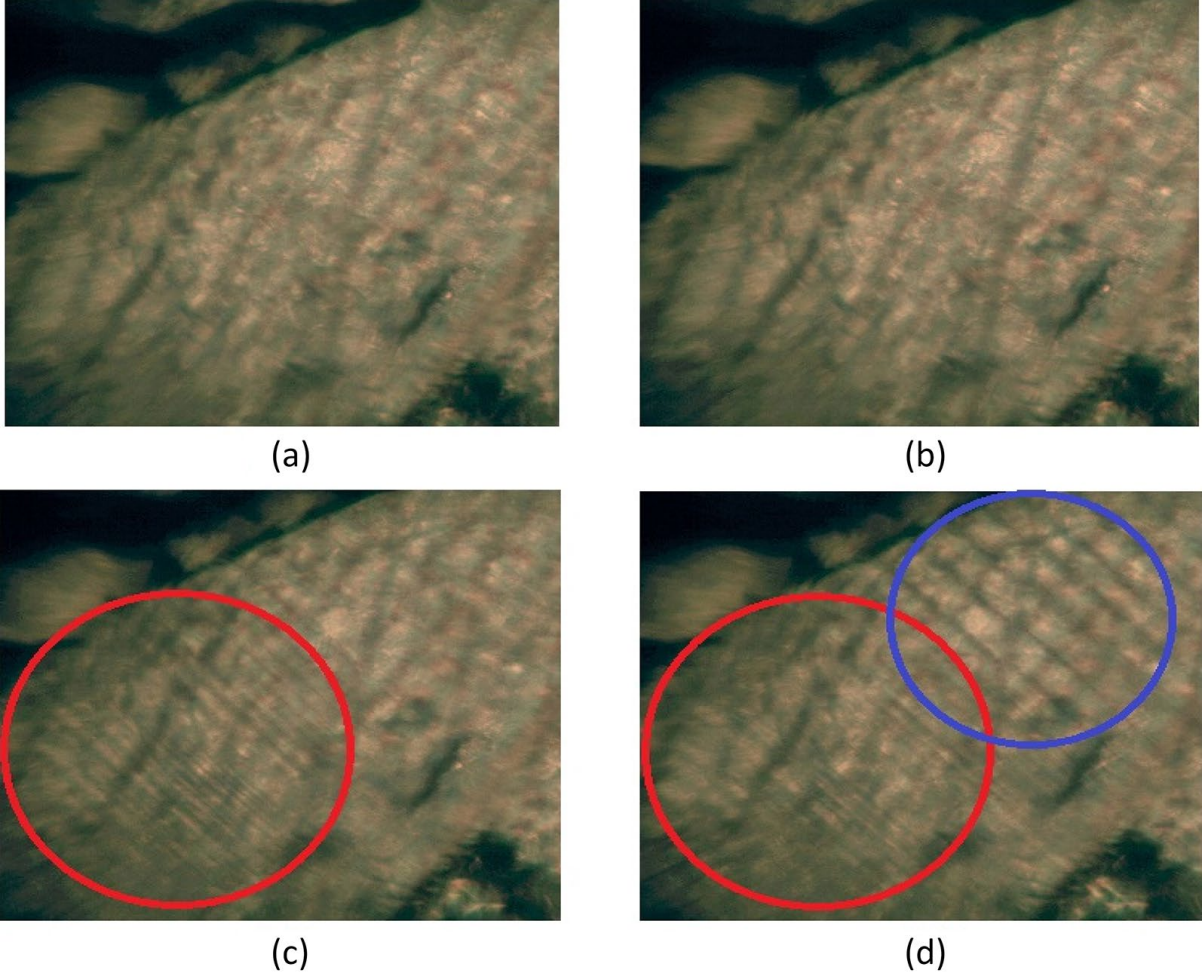
Optical polarizing microscopy images of [NH_3_(CH_2_)_5_NH_3_]CdCl_4_ at (**a**) phase III (300 K), (**b**) phase II (403 K), (**c**) phase I (420 K), and (**d**) phase I (433 K). The parallel lines represent the ferroelastic twin domain walls.

A single crystal with ferroelastic properties exhibits two or more orientation states even if mechanical stress does not exist since mechanical stress can change the existing orientation state of the single crystal. Polarized microscopy observations revealed the ferroelastic domain structures of the crystal and their changes at the phase transition temperatures, as shown in Fig. 4. The domain pattern represented by parallel lines was not observed in phases III (300 K, Fig. 4a) and II (403 K, Fig. 4b). No change in the behavior of the crystal was observed at T_C1_. However, in phase I, twinning occurred in the crystal at temperatures above T_C2_, resulting in a highly dense domain pattern indicated by the red circle (Fig. 4c). At 433 K, new domain walls indicated by the blue circles were formed next to the parallel domain walls (Fig. 4d). The phase transition at T_C2_ occurred due to the ferroelastic twin domain. The [NH_3_(CH_2_)_5_NH_3_]CdCl_4_ crystal existed in two crystallographic phases: monoclinic (*2/m*) at temperatures above 417 K, orthorhombic (*mmm*) at temperatures between 417 and 337 K, and orthorhombic (*mmm*) at temperatures below 337 K. According to Aizu^32^ and Sapriel^33^, for the transition from the *mmm* space group of the orthorhombic phase II to the *2/m* space group of the monoclinic phase I, the domain wall directions were *x* = 0 and *z* = 0. The equations of the twin domain walls was expressed as 2/*mFmmm*, corresponding to the “inverted” *mmmF2/m* instead of *mmmF2/m* as reported by Sapriel^33^.

### ^1^H MAS NMR spectrum

**Figure 5:**
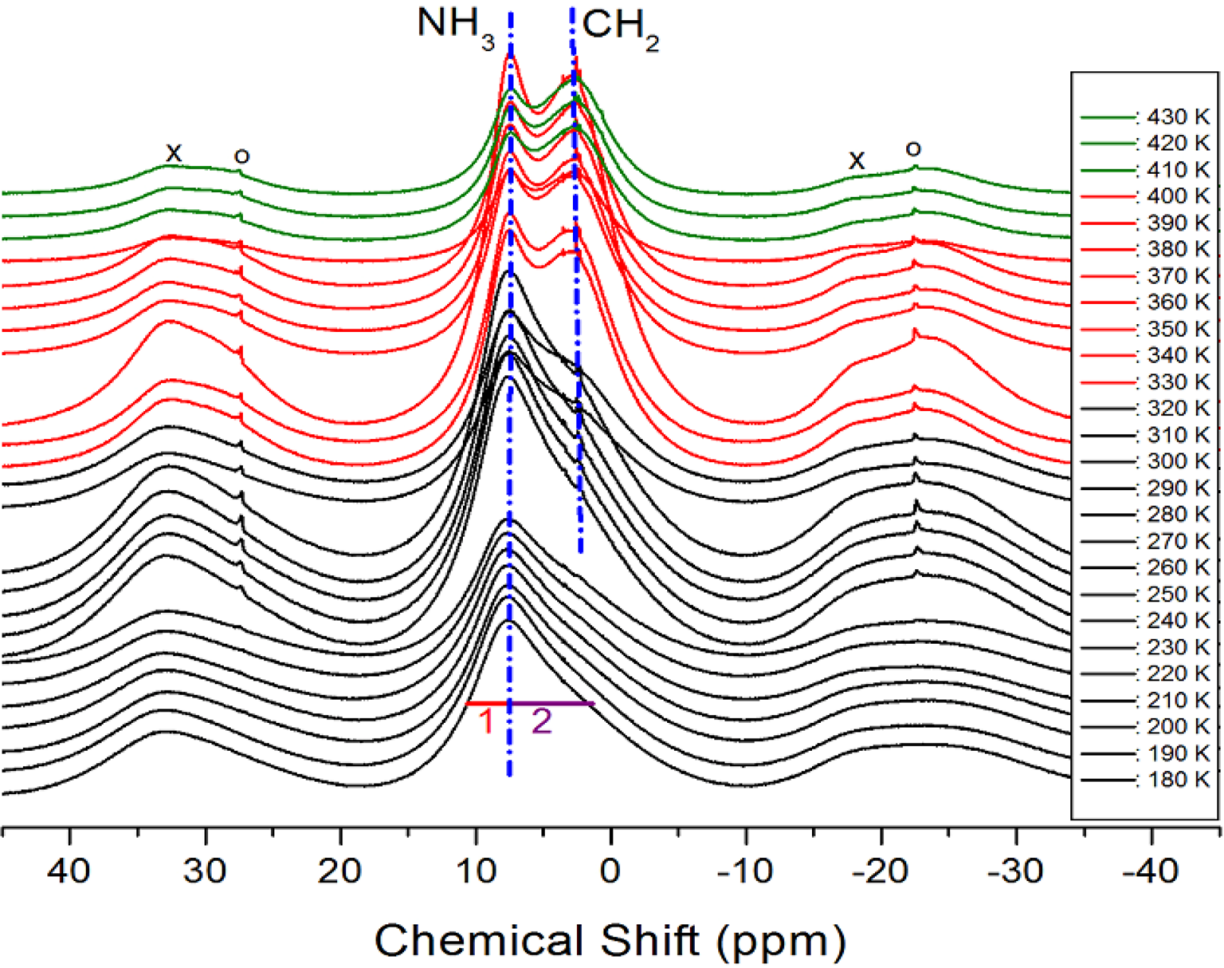
MAS ^1^H NMR spectra of [NH_3_(CH_2_)_5_NH_3_]CdCl_4_ at phases I, II, and III (olive areas: phase I, red areas: phase II, and black areas: phase III). The spinning sidebands are marked by crosses and open circles.

The ^1^H MAS NMR spectra of the [NH_3_(CH_2_)_5_NH_3_]CdCl_4_ crystal were obtained, and the ^1^H chemical shifts are shown in Fig. 5 as a function of temperature. At low temperatures, only one resonance signal was observed. These resonance signals were asymmetric due to the overlapping ^1^H lines of NH_3_ and CH_2_ in [NH_3_(CH_2_)_5_NH_3_] cations. At 180 K, a single resonance line was present at a chemical shift of 9.04 ppm. The line width and full-width at half-maximum (FWHM) at this temperature were also different from those represented as symbol “1” at 2.97 ppm and as symbol “2” at 6.07 ppm, respectively. At 330 K, which was close to T_C1_, the NMR spectrum was divided into two resonance lines, showing chemical shifts of 7.56 and 2.58 ppm for NH_3_ and CH_2_, respectively. The spinning sidebands were marked with crosses and open circles. Here, phases I, II, and III were plotted in olive, red, and black, respectively. The ^1^H chemical shifts of NH_3_ and CH_2_, presented by dotted lines in Fig. 5, were almost independent of temperature. These results suggested that the surrounding environments of ^1^H of NH_3_ and CH_2_ did not change with temperature.

### ^13^C MAS NMR spectrum

**Figure 6:**
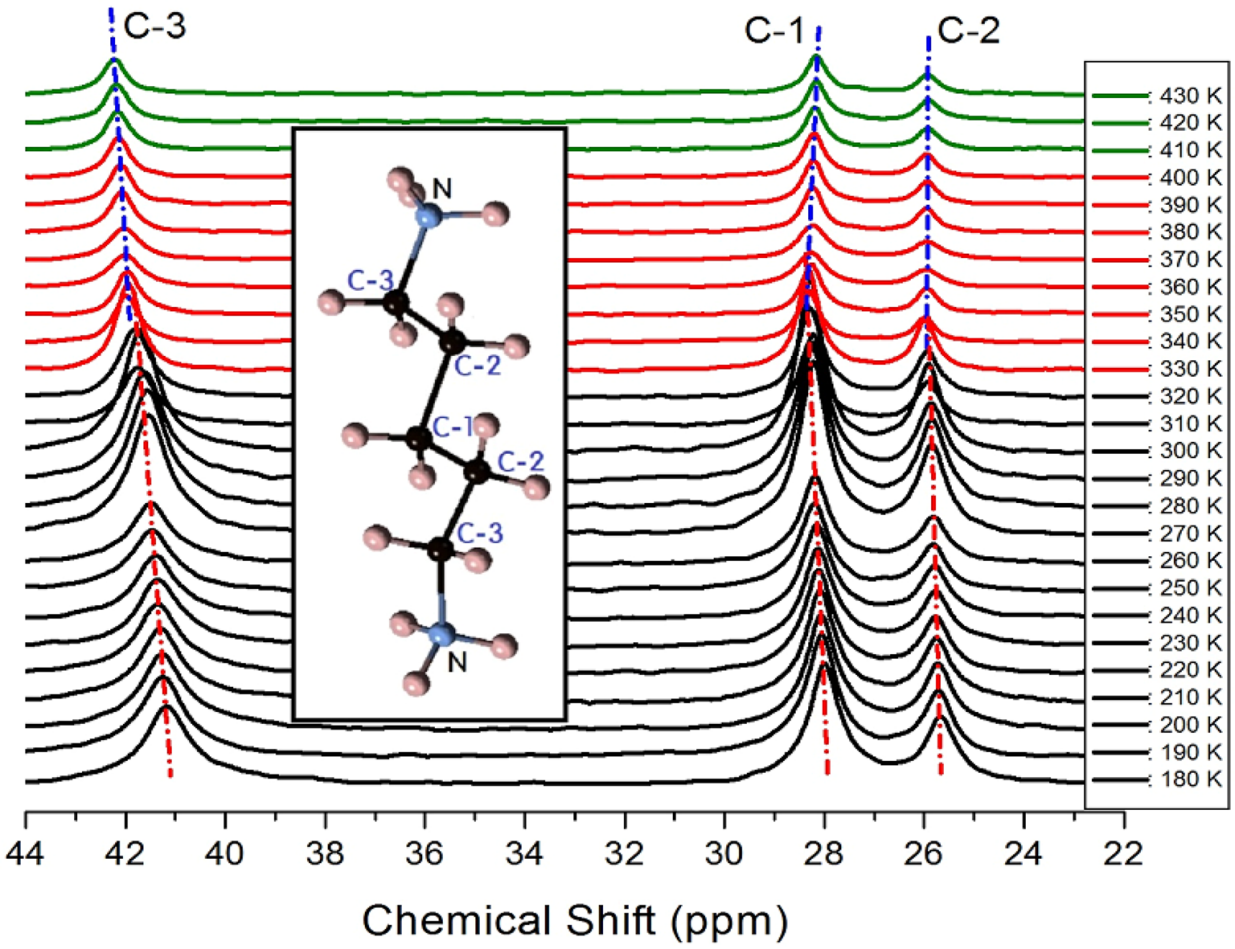
MAS ^13^C NMR spectra of [NH_3_(CH_2_)_5_NH_3_]CdCl_4_ at phases I, II, and III (olive areas: phase I, red areas: phase II, and black areas: phase III).

The ^13^C chemical shifts at increasing temperature for the in situ MAS NMR spectra are shown in Fig. 6. The TMS reference signal at 300 K recorded at 38.3 ppm was used as the standard for the ^13^C chemical shift. In the [NH_3_(CH_2_)_5_NH_3_] cation, CH_2_ located close to NH_3_ was designated as C-3, CH_2_ located at the center was designated as C-1, and CH_2_ located between C-3 and C-1 was designated as C-2. The structure of the cation for this crystal is shown in the inset of Fig. 6. At 300 K, the ^13^C chemical shifts were recorded at 28.26, 25.90, and 41.67 ppm for C-1, C-2, and C-3, respectively. The FWHM for ^13^C NMR at 300 K were 6.20, 5.72, and 9.06 ppm for C-1, C-2, and C-3, respectively. The line width of C-3 located close to N was wider than those of C-1 and C-2. The chemical shifts changed at temperatures close to T_C1_ (336 K), but not at temperatures close to T_C2_ (417 K). Below T_C1_, all ^13^C positions showed positive chemical shifts with increasing temperatures. Above T_C1_, the chemical shift of C-2 was almost independent of temperature, while the shifts in C-1 and C-3 progressed in a negative and positive direction, respectively. The results proved that below T_C1_, the surrounding environments of all ^13^C ions would change with temperature. At temperatures above T_C1_, the surrounding environments of C-2 did not change. However, the chemical shifts of C-1 and C-3 continuously changed in all temperature ranges, including T_C1_ and T_C2_.

### Static ^14^N NMR

**Figure 7:**
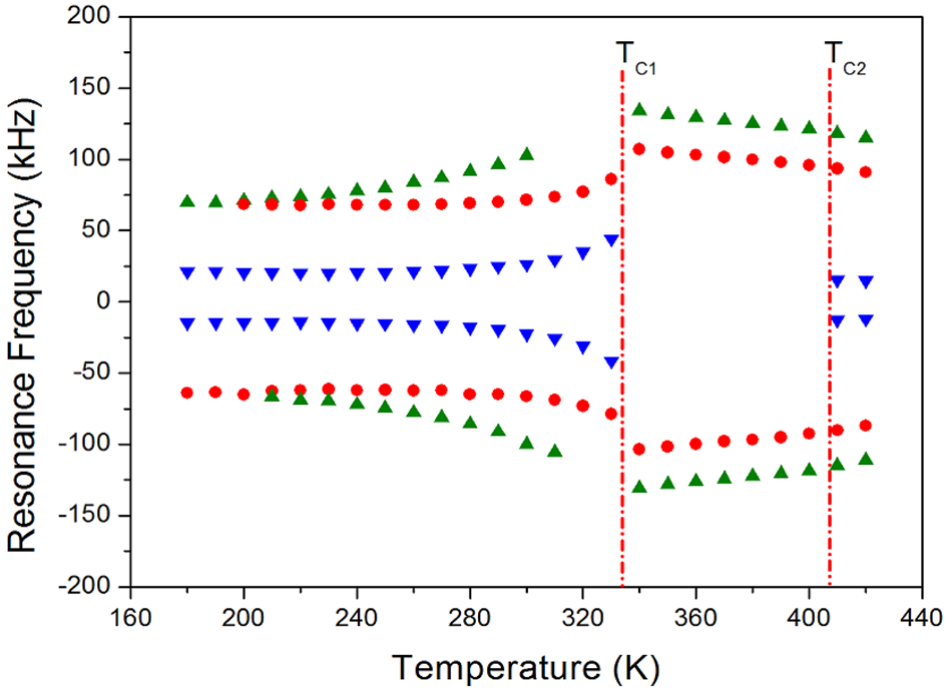
Static ^14^N NMR spectra of [NH_3_(CH_2_)_5_NH_3_]CdCl_4_ at phases I, II, and III.

The ^14^N NMR spectra of the [NH_3_(CH_2_)_5_NH_3_]CdCl_4_ single crystal in the temperature range of 180–420 K were recorded by the solid-state echo method with static NMR. Since ^14^N has quadrupole interactions with spin number I = 1, two ^14^N NMR signals were expected^34^. The ^14^N resonance frequency at increasing temperatures is shown in Fig. 7. Despite the presence of intense background noise due to the very low NMR frequency (28.90 MHz), the ^14^N spectrum was obtained without difficulty. Here, the crystal demonstrated an arbitrary direction with respect to the magnetic field. The six resonance lines of the three pairs at increasing temperatures were below T_C1_. At temperatures close to 336 K (T_C1_), the number of resonance lines and resonance frequencies of the NMR spectra showed abrupt changes. At T_C1_, a reduction from three pairs to two pairs of NMR lines was observed. At T_C2_, another pair of NMR lines reappeared. Below T_C1_, as the temperature increased, the resonance frequencies increased, and above T_C1_, as the temperature increased, the resonance frequencies decreased. At T_C2_, only the number of resonance lines changed, and the resonance frequency showed almost continuous values. Symbols with the same color indicated the same pairs of ^14^N. Changes in the ^14^N resonance frequencies due to the change in temperature were related to the changes in the crystallographic configuration of the crystal.

### ^113^Cd MAS NMR

**Figure 8:**
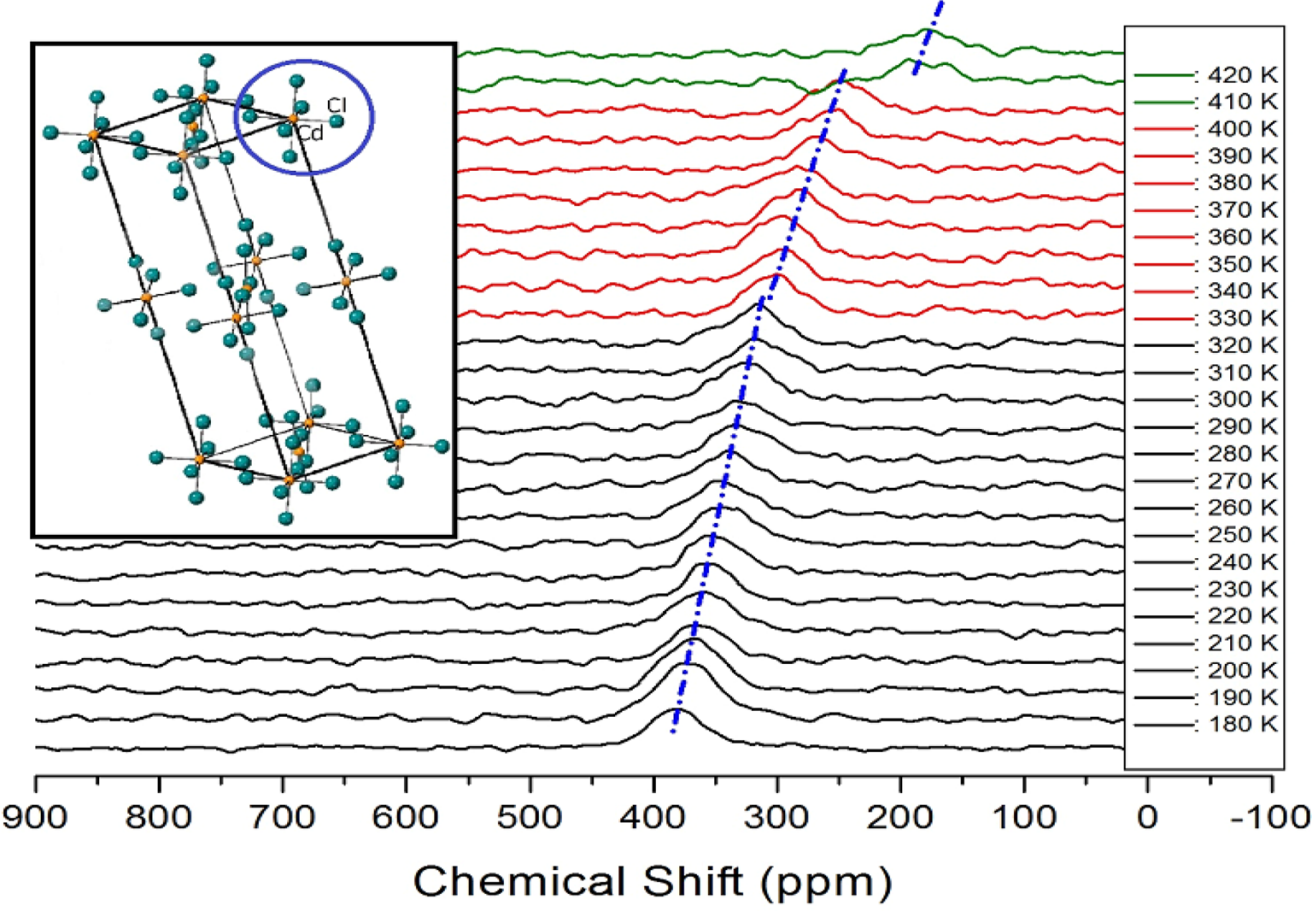
MAS ^113^Cd NMR spectra of [NH_3_(CH_2_)_5_NH_3_]CdCl_4_ at phases I, II, and III (olive areas: phase I, red areas: phase II, and black areas: phase III).

The ^113^Cd MAS NMR experiments were measured to detect the structural environments around Cd when the temperature in the CdCl_6_ anions of the [NH_3_(CH_2_)_5_NH_3_]CdCl_4_ single crystal were varied. This information was crucial to demonstrate the anion coordination environments around Cd^2+^ in CdCl_6_ using ^113^Cd NMR spectroscopy. The changes in the in situ ^113^Cd MAS NMR spectra are shown in Fig. 8. The ^113^Cd chemical shift at 300 K was 323.19 ppm. As the temperature increased, the ^113^Cd chemical shifts slightly moved in the negative direction, but these chemical shifts changed discontinuously near T_C1_ and T_C2_. In particular, more changes were observed at temperatures near T_C2_ than at temperatures near T_C1_, suggesting that temperature affected the environments around Cd. This proved that the coordination geometry of 6Cl around Cd ions in the CdCl_6_ octahedra, as shown in the inset of Fig. 8, would change at the phase transition temperatures.

### ^1^H and ^13^C spin-lattice relaxation times

**Figure 9:**
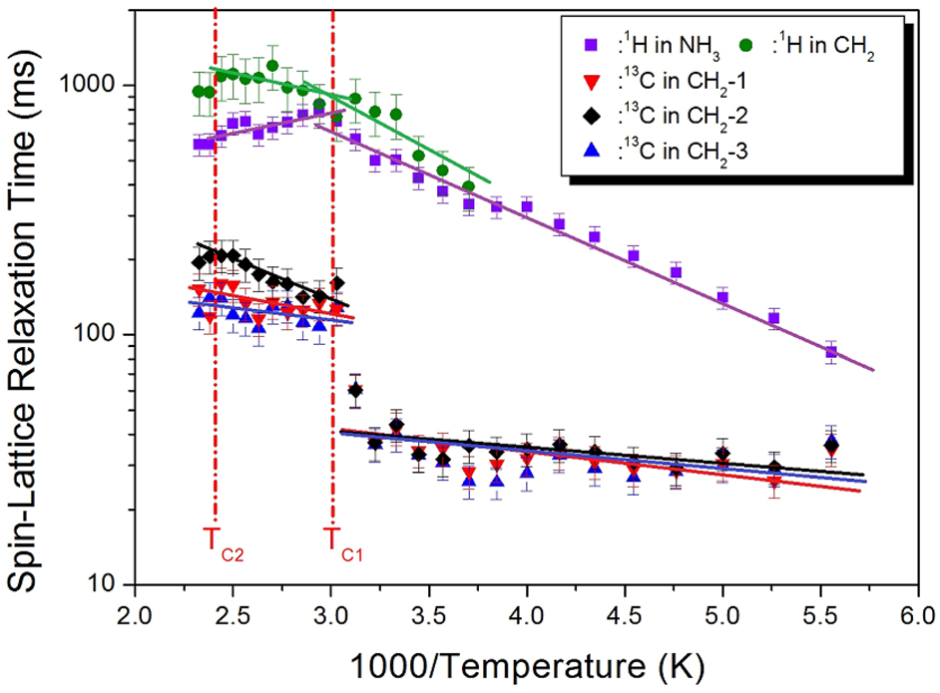
Temperature dependences of ^1^H and ^13^C NMR spin–lattice relaxation times, T_1ρ_, in [NH_3_(CH_2_)_5_NH_3_]CdCl_4_ near phase transition temperatures. Solid lines represent the activation energies.

The ^1^H MAS NMR and ^13^C MAS NMR spectra were obtained with increasing delay times, and the plot of spectral intensities against increasing delay times was expressed as an exponential function. The decay rates of the spin-locked proton and carbon magnetization are expressed as the spin-lattice relaxation time, T_1ρ_, as^34,35^:

1 $$ {\text{P}}_{{{\text{H}}({\text{C}})}} {(}\tau {)} = {\text{P}}_{{{\text{H}}({\text{C}})}} {(}0{\text{)exp}}( - \tau /{\text{T}}_{{{1}\uprho }} ), $$

where P_H(C)_(*τ*) and P_H(C)_(0) are the signal intensities for the proton (carbon) at time τ and τ = 0, respectively. The ^1^H T_1ρ_ values of NH_3_ and CH_2_ at several temperatures were determined by the slope of the logarithmic plots of intensities against delay times. From the slope of their recovery curves, the ^13^C T_1ρ_ values for C-1, C-2, and C-3 were determined. The ^1^H T_1ρ_ and ^13^C T_1ρ_ values are shown in Fig. 9 as a function of the inversed temperature. The ^1^H T_1ρ_ values increased rapidly from 100 to 1000 ms*.* While the slope of the T_1ρ_ values at temperatures near T_C1_ changed, the slope at temperatures near T_C2_ exhibited a rather continuous value. Above T_C1_, the ^1^H T_1ρ_ value for NH_3_ showed a decreasing trend. The activation energy, E_a_, values for ^1^H in NH_3_ were evaluated from the slopes (represented by the solid lines in Fig. 9) of their log T_1ρ_ versus 1000/T plots. The E_a_ values below T_C1_ were 6.65 ± 0.40 kJ/mol and 8.60 ± 2.32 kJ/mol for NH_3_ and CH_2_, respectively, while the E_a_ values above T_C1_ were 2.85 ± 0.96 kJ/mol and 3.49 ± 1.47 kJ/mol for NH_3_ and CH_2_, respectively. And, the ^13^C T_1ρ_ values below T_C1_ increased gradually with increasing temperature and then increased rapidly above T_C1_. Near T_C2_, the T_1ρ_ values were almost continuous, showing no significant changes. The E_a_ values of C-1, C-2, and C-3 below T_C_ obtained from the plot of log T_1ρ_ versus 1000/T were 1.73 ± 0.58 kJ/mol, 1.33 ± 0.49 kJ/mol, and 1.36 ± 0.76 kJ/mol, respectively. The E_a_ values of C-1, C-2, and C-3 above T_C1_ were 3.04 ± 1.38 kJ/mol, 5.57 ± 1.04 kJ/mol, and 0.97 ± 1.43 kJ/mol, respectively. The behavior of T_1ρ_ for random motions with a correlation time, τ_C_, could be described as fast- and slow-motion zones. The ^1^H and ^13^C T_1ρ_ values at low and high temperatures correspond to the fast-motion region, where ω_1_τ_C_ $\ll$ 1 and T_1ρ_^−1^ α exp(E_a_/k_B_T). In contrast, the ^1^H T_1ρ_ values in NH_3_ at high temperatures were attributed to the slow-motion region, where ω_1_τ_C_ $\gg$ 1 and T_1ρ_^−1^ α ω_1_^−2^exp(E_a_/k_B_T).

## Conclusion

The structure and phase transition temperatures of the [NH_3_(CH_2_)_5_NH_3_]CdCl_4_ crystal were confirmed using XRD and DSC. Based on the NMR analysis of the crystal, we deduced that the crystallographic surroundings of ^1^H, ^13^C, and ^14^N in the cation at temperatures close to T_C1_ changed, whereas that of ^113^Cd in the anion at temperatures close to T_C1_ and T_C2_ exhibited significant changes. The changes in the NMR chemical shifts near T_C1_ and T_C2_ also suggested that the N–H···Cl hydrogen bond was affected.

On the other hand, the T_1ρ_ values of ^1^H in NH_3_ changed from fast to slow motion near T_C1_. The T_1ρ_ values of ^13^C in CH_2_ increased rapidly at T_C1_, and the E_a_ values for ^13^C were more flexible at low temperatures than at high temperatures. By evaluating the T_1ρ_ values, we deduced that the effect on the molecular motion was effective at high temperatures.

Consequently, the phase transition at 336 K was associated with the change in the N–H···Cl bond due to the change in the coordination geometry of Cl around Cd in the CdCl_6_ anion. The phase transition at 417 K was related to the ferroelastic phase transition attributed to the twin domains.

The thermodynamic properties, ferroelastic domain walls, coordination geometries, and molecular motions of [NH_3_(CH_2_)_5_NH_3_]CdCl_4_ in this study are thought to be helpful in the study of hybrid perovskite types for their various applications in batteries and fuel cells.
